# DOES THE NEWS MEDIA FOSTER A HOSTILE ENVIRONMENT FOR OLDER ADULTS: A CONTENT ANALYSIS OF NEWS ARTICLES

**DOI:** 10.1093/geroni/igz038.3470

**Published:** 2019-11-08

**Authors:** Erfei Zhao

**Affiliations:** University of Southern California, Los Angeles, California, United States

## Abstract

The media has become increasingly more agenda-driven and politically biased in recent years. This research explores whether the major news outlets fosters an environment of negative sentiment towards the older adult population over a 4-year-period from 2016-2019. The paper uses a content analysis approach. 199 news articles that include keywords such as "older adults", "seniors" or "elderly" are randomly selected among those published in the first week of March in 2016, 2017, 2018 and 2019 from the online archives. Main attitudes towards the older adults are extracted and categorized. In the 4-year span, regression analysis shows that negative attitudes are apolitical and are found in almost two-thirds (64%) of the 199 news articles in both liberal and conservative news outlets, suggesting that the current media creates a ubiquitously hostile environment for older adults. However, more liberal news outlets tend to produce more articles with positive messages about the older adults (p<0.05), and more conservative news outlets tend to create more content that reinforces negative stereotypes of older adults as being vulnerable and less productive (p<0.01). The high percentage of negative attitudes and the ubiquitous negative attitudes in news organizations, in spite of their political leaning, suggests that the media environment has some influence on the news content. Political biases of the organization are associated to numbers of articles with positive messages and negative stereotyping. Promoting professional development and self-regulating mechanisms in journalism could aid in reducing ageism perpetuated in the media.

